# Effect of a single dose of insulin glargine/lixisenatide fixed ratio combination (iGlarLixi) on postprandial glucodynamic response in Japanese patients with type 2 diabetes mellitus: A phase I randomized trial

**DOI:** 10.1111/dom.13757

**Published:** 2019-05-24

**Authors:** Megumi Inoue, Martin Lorenz, Hideya Muto, Roland Wesch, Yasuhiro Hashimoto

**Affiliations:** ^1^ SOUSEIKAI PS Clinic Fukuoka Japan; ^2^ Research & Development, Sanofi‐Aventis Deutschland GmbH Frankfurt Germany; ^3^ Research & Development, Sanofi K.K. Tokyo Japan

**Keywords:** basal insulin, GLP‐1 analogue, glycaemic control, pharmacodynamics, pharmacokinetics, phase I–II study

## Abstract

This report describes novel clinical data assessing the pharmacodynamics of insulin glargine/lixisenatide (iGlarLixi) compared with placebo and insulin glargine alone, to determine pharmacokinetics of lixisenatide, and to assess safety of iGlarLixi in Japanese people with type 2 diabetes mellitus (T2DM). In a single‐centre, open‐label, randomized, placebo‐controlled cross‐over study, participants received subcutaneous iGlarLixi 5 U/5 μg and 10 U/10 μg, placebo, and 5 U insulin glargine. The primary endpoint was area under the postprandial plasma glucose (PPG) curve (AUC_0–2h_). A total of 20 participants completed all study periods. iGlarLixi 5 U/5 μg and 10 U/10 μg reduced mean PPG dose‐dependently compared with placebo and insulin glargine 5 U. Both combinations significantly reduced PPG‐AUC_0–2h_ dose‐dependently compared with placebo (least squares mean difference −7.48 mmol h/L for 5 U/5 μg, −10.75 mmol h/L for 10 U/10 μg; *P* < 0.0001). iGlarLixi 5 U/5 μg reduced PPG‐AUC_0–2h_ significantly compared with insulin glargine 5 U (−0.76 mmol h/L; *P* < 0.0001). No symptomatic hypoglycaemia occurred during the study. iGlarLixi single subcutaneous injections significantly and dose‐dependently reduced PPG compared to placebo or insulin glargine in Japanese participants with T2DM. iGlarLixi was safe and well tolerated, and would be expected to provide the 24‐hour plasma glucose‐lowering effects of insulin glargine and the postprandial antihyperglycaemic effects of lixisenatide.

## 1 INTRODUCTION

The American Diabetes Association, the European Association for the Study of Diabetes and the Japan Diabetes Society currently recommend rapid‐acting insulin analogues as needed to avoid postprandial hyperglycaemia in people with type 2 diabetes mellitus (T2DM)^1^, ^2^; however, such additions can complicate therapy for patients, particularly if multiple injections are required, and can be associated with hypoglycaemic events and weight gain, leading to low adherence and poor compliance.^3^ Alternative treatment options are needed to address both fasting and postprandial blood glucose effectively.

A new combination product, iGlarLixi, delivers the action and effects of both basal insulin and a glucagon‐like‐peptide‐1 (GLP‐1) receptor agonist in a single injection. The iGlarLixi combination product, insulin glargine (100 U/mL) and lixisenatide (100 μg/mL), is administered once daily by subcutaneous injection. Insulin glargine, an analogue of human insulin, provides a sustained 24‐hour supply of basal insulin with once‐daily single‐dose subcutaneous injection.^3^ Lixisenatide is a once‐daily GLP‐1 receptor agonist used for glycaemic control in a number of countries, including Japan. Lixisenatide slows gastric emptying and prolongs absorption of meal‐derived glucose, reducing the rate at which meal‐derived glucose is absorbed and circulated, and controlling postprandial plasma glucose (PPG) excursions.^4^, ^5^, ^6^ Insulin glargine and lixisenatide improve fasting and postprandial glucose levels complimentarily, and can be used effectively together in separate injections. The iGlarLixi formulation is expected to provide a benefit–risk ratio superior to either insulin glargine or lixisenatide alone.

In Europe, the United States, and a number of other countries, iGlarLixi has already been approved at ratios of 2:1 (2 units of insulin glargine to 1 μg lixisenatide) and 3:1 (3 units to 1 μg) for once‐daily subcutaneous injection. The phase III studies LixiLan‐L and LixiLan‐O investigated the efficacy and safety of iGlarLixi versus therapy with either lixisenatide or insulin glargine; results indicate that the combination product is both effective and safe in people with T2DM.^7^, ^8^ In Japan, the product has been modified to a 1:1 combination (1 unit of insulin glargine to 1 μg lixisenatide), which better suits Japanese patients' needs. This is because Japanese people tend to require a lower insulin dose as they usually have lower body weight and body mass index (BMI) than their Western counterparts.^9^ Differences in BMI explain the differences seen in insulin sensitivity and ß‐cell responsiveness between Japanese people and people from Western countries^10^, ^11^, ^12^ and the need for a lower insulin dose in Japanese people.

In the present paper, we report the findings from a single‐dose study to investigate the pharmacodynamic (PD) effects of two doses of iGlarLixi versus placebo and versus insulin glargine alone, to determine the pharmacokinetic (PK) effects of lixisenatide, and to assess the safety and tolerability of iGlarLixi in Japanese people with T2DM.

## 2 METHODS

### 2.1 Study design

This was a phase I, single‐centre, randomized, open‐label, placebo‐controlled, four‐sequence, four‐period, four‐treatment crossover, single‐dose study.

Inclusion and exclusion criteria are provided in Appendix S1. Participants were randomized to one of four sequences, receiving single subcutaneous injections of 5 U insulin glargine/5 μg lixisenatide (iGlarLixi 5 U/5 μg), 10 U insulin glargine/10 μg lixisenatide (iGlarLixi 10 U/10 μg), insulin glargine 5 U and placebo in a randomized order in four treatment periods, under fasted conditions. There was a washout period of 7 to 14 days between dose administrations.

Participants provided written informed consent before any study procedures. The study protocol was approved by the institutional review board at SOUSEIKAI Hakata Clinic and was conducted in accordance with the Declaration of Helsinki and Good Clinical Practice guidelines. The study was registered with clinical trials number NCT02713477.

### 2.2 Assessments

The primary endpoint was PPG area under the concentration–time curve (AUC) from the time of breakfast start until 2 hours later (PPG‐AUC_0–2h_). The secondary endpoints are described in Appendix S1.

### 2.3 Statistical methods

The PD analyses were performed using the evaluable PD population. PPG–AUC_0–2h_ was analysed using a linear mixed‐effects model with treatment group, sequence and treatment period as fixed effects, patient‐within‐sequence as a random effect, and baseline value as a covariate. The least squares (LS) mean differences were calculated between iGlarLixi groups and placebo, between the two iGlarLixi groups, and between iGlarLixi 5 U/5 μg and insulin glargine. The corresponding 95% confidence intervals and *P* values were calculated within the linear mixed‐effect model framework. *P* values < 0.05 were taken to indicate statistical significance. No adjustment for multiplicity was performed. The sample size calculation and statistical analysis methods for the secondary endpoints are described in Appendix S1.

## 3 RESULTS

### 3.1 Participants

A total of 20 people with T2DM were randomized and treated. All participants completed all four treatment periods. All randomized partcipants were included as applicable in the evaluable PD, PK and safety populations (Figure S1). Baseline demographics and other characteristics are summarized in Table S1.

### 3.2 Pharmacodynamics

**Figure 1:**
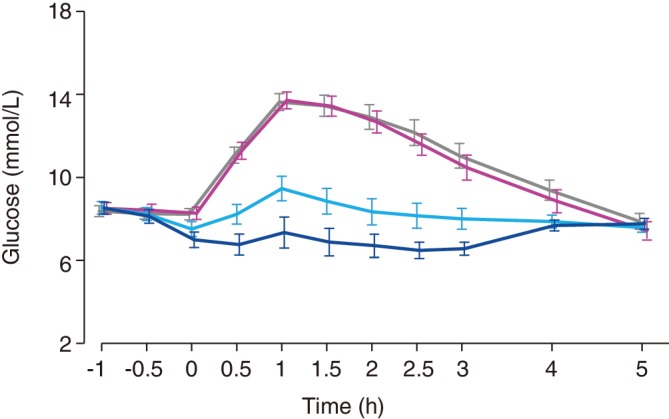
Pharmacodynamic results of insulin glargine/lixisenatide fixed ratio combination treatment: changes in serum and plasma glucose levels over time. ^†^Time is shown in reference to the start of breakfast (0 hour on the *x*‐axis). Breakfast was served 1 hour after administration of the study drug

For the primary endpoint, iGlarLixi (5 U/5 μg and 10 U/10 μg) demonstrated a clear dose‐dependent reduction in plasma glucose concentrations compared to placebo and insulin glargine. PPG‐AUC_0–2h_ was significantly lower for both iGlarLixi doses than for placebo, and this difference was dose‐dependent. In addition, iGlarLixi 5 U/5 μg provided significantly greater reduction in PPG‐AUC_0–2h_ than insulin glargine 5 U (Figure 1 and Table 1).

**Table 1:** Pharmacodynamic results of insulin glargine/lixisenatide fixed ratio combination treatment

	iGlarLixi 5 U/5 μg	iGlarLixi 10 U/10 μg
	(n = 20)	(n = 20)
PPG‐AUC_0 − 2h_, mmol h/L		
LS mean difference (SE) vs placebo^a^	−7.48 (0.65)	−10.75 (0.65)
95% CI	−8.78, −6.17	−12.06, −9.45
*P*	<0.0001	<0.0001
LS mean difference (SE) vs iGlarLixi 5 U/5 μg^b^		−3.28 (0.65)
95% CI		−4.58, −1.97
*P*		<0.0001
LS mean difference (SE) vs insulin glargine^c^	−7.30 (0.65)	
95% CI	−8.60, −6.00	
*P*	<0.0001	
PPG‐C_max_, mmol/L		
LS mean difference (SE) vs placebo^a^	−4.21 (0.37)	−4.97 (0.37)
95% CI	−4.96, −3.47	−5.71, −4.23
*P*	<0.0001	<0.0001
LS mean difference (SE) vs iGlarLixi 5 U/5 μg^b^		−0.76 (0.37)
95% CI		−1.50, −0.02
*P*		0.0454
LS mean difference (SE) vs insulin glargine^c^	−3.88 (0.37)	
95% CI	−4.62, −3.14	
*P*	<0.0001	
PPG‐t_max_, h		
Mean (SE)	1.93 (0.321)	3.08 (0.399)

*Note*: The linear mixed‐effects model that was used included treatment group, sequence and treatment period as fixed effects, participant‐within‐sequence as a random effect, and the corresponding baseline all parameters value (−1 hour for time for pharmacodynamics) as a covariate.

Abbreviations: AUC, area under the curve; CI, confidence interval; C_max_, maximum concentration; iGlarLixi, insulin glargine/lixisenatide fixed ratio combination; LS, least squares; PPG, postprandial plasma glucose; SE, standard error; t_max_, time to maximum concentration.

a Difference: iGlarLixi 5 U/5 μg − placebo and iGlarLixi 10 U/10 μg − placebo, respectively.

b Difference: iGlarLixi 10 U/10 μg − iGlarLixi 5 U/5 μg.

c Difference: iGlarLixi 5 U/5 μg − insulin glargine.

In secondary endpoints, changes in maximum PPG concentration corresponded to those seen for the primary endpoint. LS mean differences were statistically significant between the combination and placebo, between the two dose levels of the combination, and between iGlarLixi 5 U/5 μg and insulin glargine 5 U. Mean plasma glucose concentrations peaked approximately 1 hour after breakfast start (at 2 hours post‐dose) for placebo and insulin glargine (Figure 1). The mean PPG‐time to maximum concentration was prolonged for iGlarLixi, with a dose‐dependent delay compared to placebo (Table 1).

The results of other secondary endpoints and PK variables are provided in Figures S2 and S3, Tables S2–S4, and Appendix S1.

### 3.3 Safety

Three participants experienced mild nausea, and there were three instances of asymptomatic hypoglycaemia, for iGlarLixi 10 U/10 μg. No other treatment‐emergent adverse events were reported during the study. No symptomatic hypoglycaemia occurred during the study. The safety details are provided in Table S5 and in Appendix S1.

## 4 DISCUSSION

In the present study in Japanese people with T2DM, iGlarLixi decreased plasma glucose concentrations dose‐dependently compared to placebo and insulin glargine alone. PPG reduction was significantly greater with iGlarLixi than with placebo and with low‐dose insulin glargine, and the iGlarLixi‐induced reduction of PPG‐related variables was dose‐dependent. This reduction was attributed to the lixisenatide component in iGlarLixi, which delays gastric emptying, inhibits glucagon secretion, and suppresses glucose elevation.^5^, ^6^ Because plasma glucose was not elevated in the iGlarLixi groups, the plasma insulin level did not change greatly with food intake, and endogenous insulin secretion (as measured by C‐peptide level) was not increased (Figure S2 and Table S2). The increase in insulin levels from just after administration, both in the iGlarLixi and insulin glargine groups, was attributed to the effects of exogenous insulin. These findings are aligned with previous clinical pharmacology studies in healthy people and people with T2DM, showing that lixisenatide effectively slows gastric emptying and prolongs absorption of meal‐derived glucose, and thus could blunt postprandial glucose excursions.^5^, ^6^

Single‐dose subcutaneous injection of iGlarLixi was safe and well tolerated. Adverse events were consistent with previously reported safety profiles for lixisenatide and insulin glargine. Instances of asymptomatic hypoglycaemia in the high‐dose iGlarLixi group were attributed to PPG reduction with high‐dose iGlarLixi. There were three instances of nausea for iGlarLixi 10 U/10 μg in the present single‐dose study. The relatively low level of gastrointestinal symptoms may be attributable to two points: first, the study involved only single‐dose administration; second, the lixisenatide dose was lower than the usual maintenance dose. There were also three instances of asymptomatic hypoglycaemia for iGlarLixi 10 U/10 μg, but no symptoms developed. No other safety‐related issues were noted (Table S5).

The ratio of the insulin glargine/lixisenatide combination outside Japan is 2:1 or 3:1. In the present study, the combination was formulated at a ratio of 1:1 to address the lower required insulin dose because of the lower body weight and BMI of Japanese people. Results suggest no notable concerns related to PD or PK variables, or safety, and the combination significantly reduced PPG compared to placebo or insulin glargine.

Of particular note, results indicated the efficacy of lixisenatide 5 μg. The usual starting dose for lixisenatide is 10 μg, and the usual maintenance dose is 20 μg; no data have previously been available on the efficacy of the considerably lower dose of 5 μg. This study compared iGlarLixi 5 U/5 μg with insulin glargine 5 U, which also provided information on the additional effects of lixisenatide 5 μg in treatment with the combination product iGlarLixi. At that dose, iGlarLixi provided significantly greater reduction in PPG than was obtained from insulin glargine 5 U alone, clearly indicating the PPG‐lowering action of lixisenatide 5 μg.

Asian people with T2DM face the risk of hypoglycaemia, weight gain, difficulties in reducing PPG, and the complexities of subcutaneous injection, dose titration, and regular self‐monitoring of blood glucose associated with basal insulin therapy. The combination product iGlarLixi could therefore address a medical need in Japanese patients to provide both the sustained FPG‐lowering effects of insulin glargine and the complimentary PPG‐lowering effects of lixisenatide in a single convenient once‐daily injection rather than several injections throughout the day. Lixisenatide is also expected to inhibit weight gain, which may compensate for some of the weight increase associated with insulin therapy.

There have been numerous reports of previous clinical trials of iGlarLixi; however, the present study has three aspects that differentiate it from previous studies. First, the 1:1 ratio of drugs in the formulation differs from that used in previous non‐Japanese studies. Second, previous PK/PD studies of iGlarLixi were conducted in people with type 1 diabetes and healthy adults. To our knowledge, this is the first study that has focused on PK/PD characteristics in Japanese people with T2DM. Third, the present study investigated patient response to low‐dose (5 U/5 μg) iGlarLixi.

The present study had a limited number of participants and involved only single‐dose administration. The ongoing phase III clinical studies will provide further information to evaluate the efficacy and safety of repeated‐dose administration in Japanese people with T2DM.

In conclusion, single‐dose subcutaneous administration of iGlarLixi 5 U/5 μg or 10 U/10 μg resulted in significantly greater reduction in PPG than either placebo or low‐dose insulin glargine in Japanese people with T2DM. This PPG reduction was found to be dose‐dependent. Single doses of iGlarLixi were safe and well tolerated, and the combination product can be expected to provide effective 24‐hour plasma glucose‐lowering effects from the fasting glucose benefits of insulin glargine, together with the postprandial antihyperglycaemic effects of lixisenatide.

## CONFLICT OF INTEREST

M.I. reports no conflict of interest. M.L. and R.W. are employed by Sanofi‐Aventis Germany GmbH. H.M. and Y. H. are employed by Sanofi K.K. Japan.

## AUTHOR CONTRIBUTIONS

M.L., H.M. and R.W. contributed to the study design. M.I. contributed to the conduct/data collection. Y.H. contributed to analysis. All authors contributed to the interpretation of data and the writing, reviewing, and editing of the manuscript, and had final responsibility for approving the published version of the manuscript.

## DATA AVAILABILITY

The data, analytical methods, and study materials will not be made available to other researchers at this point.

## Supporting information


**Appendix S1.** Supplemental Appendix.

**TABLE S1.** Patient characteristics.
**Table S2.** Results for additional PD parameters of iGlarLixi.
**Table S3.** Results for PK parameters of lixisenatide.
**Table S4.** Dose proportionality assessment of lixisenatide
(iGlarLixi 5 U/5 μg vs iGlarLixi 10 U/10 μg).
**Table S5.** Summary of adverse events.
**Figure S1.** Patient disposition.
**Figure S2.** Changes in serum and plasma parameters for pharmacodynamics: insulin, C‐peptide and plasma glucagon.
**Figure S3.** Changes in plasma concentration of lixisenatide for pharmacokinetics.
