# Supplementary material for: Effect of a single dose of insulin glargine/lixisenatide fixed ratio combination (iGlarLixi) on postprandial glucodynamic response in Japanese patients with type 2 diabetes mellitus: A phase I randomized trial
Source: Diabetes Obes Metab. 2019 May 24;21(8):2001–5. doi: 10.1111/dom.13757 (PMC6771557; doi:10.1111/dom.13757)
Supplement: Supplementary file 1 — Appendix S1. Supplemental Appendix. [file DOM-21-2001-s001.docx]

**Supplemental Appendix**

**Effect of single dose of insulin glargine/lixisenatide fixed ratio combination (iGlarLixi) on postprandial glucodynamic response in Japanese patients with type 2 diabetes mellitus: A phase 1 randomized trial**

**Details of study design, patient treatment, data assessment, statistical methods, and results**

**METHODS**

***Study design and patient treatment***

*Inclusion and exclusion criteria*

The study subjects were Japanese male or female patients aged 20 to 75 years with a diagnosis of type 2 diabetes mellitus (T2DM) at least 1 year before screening, and having body mass index of ≤35.0 kg/m^2^, glycated hemoglobin (HbA1c) level between 7.0% and 10.0% (inclusive), fasting plasma glucose (FPG) level between 110 mg/dL and 250 mg/dL (inclusive), and fasting C-peptide level of ≥0.6 ng/mL. Exclusion criteria included diabetes other than T2DM, pregnancy or breast-feeding in women, history of hypoglycemia unawareness, and current or past medical history or medication use that might adversely affect the patient’s safety or influence the accuracy of study findings. The use of any other injectable antidiabetic or hypoglycemic agent, any oral antidiabetic or hypoglycemic agent other than metformin, systemic glucocorticosteroids, and weight loss drugs was prohibited during the study.

*Treatment*

The study drugs were 5U/5µg and 10U/10µg iGlarLixi, placebo, and insulin glargine 5U. The iGlarLixi and placebo were supplied in 3-mL cartridges, and insulin glargine was provided in 3-mL disposable pens (Lantus® SoloStar®). The study drugs were administered subcutaneously to patients under fasted conditions at about 8:00 AM on day 1 of each treatment period. Patients had a standardized breakfast 1 hour after the study drug administration.

Blood samples for pharmacodynamics (PD) assessment were collected at 1 and 0.5 hours before the start of breakfast, and 0, 0.5, 1, 1.5, 2, 2.5, 3, 4 and 5 hours after the start of breakfast. Blood samples to measure plasma lixisenatide concentrations were collected for treatment with 5U/5µg and 10U/10 µg iGlarLixi only, just prior to administration and 0.5, 1, 1.5, 2, 2.5, 3, 4, 5, 6, 8, and 10 hours after administration.

***Assessments***

*Secondary endpoints for pharmacodynamics*

Secondary endpoints for PD included maximum postprandial plasma glucose (PPG) (PPG-C_max_), time of PPG-C_max_ (PPG-t_max_) and PPG-AUC_0-5_. In addition, serum insulin, serum C-peptide, and plasma glucagon concentrations were analyzed as secondary endpoints, and appropriate PD parameters such as AUCs were evaluated as applicable.

*Pharmacokinetics*

The following pharmacokinetics (PK) parameters for lixisenatide were evaluated from plasma lixisenatide concentrations using standard noncompartmental methods: maximum observed concentration (C_max_); time to reach C_max_ (t_max_); AUC extrapolated to infinity (AUC); time corresponding to the last concentration above the limit of quantification (t_last_); AUC from time zero to the real time t_last_ (AUC_last_); terminal half-life (t_1/2_); mean apparent clearance (CL/F), and mean apparent volume of distribution (Vss/F).

*Safety*

Patient safety was monitored from adverse events (AEs) spontaneously reported by a patient or noted by an investigator, including hypoglycemic events. All AEs, regardless of seriousness or relationship to the study drug, were recorded in each patient’s records from the time the informed consent form was signed. Treatment emergent AEs (TEAEs) were defined as AEs that occurred or worsened or became serious during the treatment phase.

***Statistical analysis***

*Sample size calculation*

Based on previous exploratory analysis (NCT01572649) in non-Japanese adult patients with T2DM for a single dose of placebo, lixisenatide 5μg, or lixisenatide 10μg, a PPG-AUC_0-2_ pairwise treatment difference of 85, 90 and 95 mg⋅hr/dL and within-subject standard deviation between 60 and 80 mg⋅hr/dL were assumed. Under most assumptions, with a sample size of 16 evaluable patients (4 patients per sequence), this 4 x 4 crossover design generally has at least 90% power to detect a treatment difference using a 2-group t-test with a 2-sided significance level of 0.05. Allowing for dropouts, 20 patients (5 patients per sequence) were enrolled to have 16 evaluable patients available for PD evaluation in this study.

*Analysis methods for the secondary endpoints for pharmacodynamics, pharmacokinetics, and safety*

The evaluable PD population was defined as patients from the full analysis PD population (1) who had completed all four treatment periods in compliance with the protocol and (2) for whom reliably evaluable blood samples were available. In addition to applying the linear mixed effects model to PPG-AUC_0-2_ and the least squares method to iGlarLixi-related differences in the study groups, other appropriate PD parameters for PPG and secondary endpoints were also analyzed using the same statistical model as for PPG-AUC_0-2_, with the corresponding baseline value as covariate. Descriptive statistics were provided by treatment group for PPG-t_max_,

The PK values for lixisenatide were analyzed using the evaluable PK population. That population was defined as patients from the full analysis PK population (1) who had completed the two fixed-ratio combination treatments in compliance with the protocol and (2) for whom reliably evaluable blood samples were available. The PK parameters of lixisenatide were summarized by treatment group, using descriptive statistics. The log-transformed PK parameters of lixisenatide were compared between the two fixed ratio combination groups using the linear mixed effect model with fixed terms for treatment group, sequence, treatment period and a random term for a patient-within-sequence. The point estimate and 90% CI for the ratio of the treatment mean ([iGlarLixi 10U/10µg] / [iGlarLixi 5U/5µg]) were converted by antilog transformation to the original scale.

Evaluation of safety was based on the review of individual values and descriptive statistics. All safety analyses were performed in the safety population. Individual values were flagged for potentially clinically significant abnormalities (PCSAs) if appropriate.

**RESULTS**

***Additional secondary endpoints for pharmacodynamics***

Effects for PPG-AUC_0-5_ were similar to those for the primary endpoint. LS mean differences in PPG-AUC_0-5_ were statistically significant between the combination and placebo (-14.88 mmol⋅hr/dL for 5U/5μg; *P*< .0001: -20.55 mmol⋅hr/L for 10U/10μg; *P*< .0001) and between the 2 dose levels of the combination (-5.67 mmol⋅hr/L; *P*= .0001), and also between iGlarLixi 5U/5μg and insulin glargine 5U alone (-12.90 mmol⋅hr/L; *P*< .0001) (Table S2).

Mean preprandial serum insulin concentration increased in a dose-dependent manner following the administration of iGlarLixi 5U/5μg and 10U/10μg in comparison to insulin glargine and placebo, and postprandial increases were suppressed for 3 hours after breakfast. The LS mean values of insulin AUC_0-2_ were comparable between the 2 combinations and placebo, with no significant differences among the 3 groups. The difference in insulin AUC_0-2_ between iGlarLixi 5U/5μg and 5U insulin glargine was statistically significant (-38.75 pmol⋅hr/L; *P*= .0263). Serum insulin levels 5 and 6 hours after administration of iGlarLixi were higher than in the insulin glargine and placebo groups, and this difference was dose-dependent. When compared with premeal levels, mean serum insulin concentration trended dose-dependently lower with iGlarLixi than with placebo for up to 3 hours after the meal (Figure S2, Table S2).

After administration, the mean serum C-peptide concentration increased for both doses of iGlarLixi before breakfast. After breakfast, serum C-peptide increased sharply in the insulin glargine 5U and placebo groups. This increase was less pronounced in the two iGlarLixi groups; the amount of increase was inversely dose-dependent. Serum C-peptide AUC_0-2_ was significantly reduced with iGlarLixi 10U/10μg in comparison to placebo (-0.33 nmol⋅hr/L;*P*= .0007) and also in comparison to 5U/5μg (-0.26 nmol⋅hr/L; *P*= .0065) (Figure S2, Table S2).

For both iGlarLixi dose levels, the mean concentration of plasma glucagon (AUC_0-2_) was lower than for placebo. This difference was significant for iGlarLixi 5U/5μg (-23.88 ng⋅hr/L; *P*= .0061) but did not reach significance for 10U/10μg (-5.08 ng⋅hr/L; *P*= .5515) (Figure S2, Table S2). The mean plasma glucagon AUC_0-2_ was also significantly lower for iGlarLixi 5U/5 μg than for insulin glargine 5U.

***Pharmacokinetics***

Changes in plasma concentration of lixisenatide for PK were shown in Figure S3. Investigation of pharmacokinetics showed that, after subcutaneous injection of iGlarLixi, lixisenatide was absorbed from the injection site at a median t_max_ of 2.50 hours for 5U/5μg and 2.00 hours for 10U/10μg. Mean C_max_ was 51.6 pg/mL and 110 pg/mL, respectively, and AUC_last_ was 249 pg⋅hr/mL and 556 pg⋅hr/mL, respectively (Table S3). The PK parameters for lixisenatide (C_max_, AUC_last_, and AUC_0-2_) increased dose-proportionally (Table S4). The CL/F of lixisenatide was 19.9 L/hr for iGlarLixi 5U/5μg and 18.2 L/hr for 10U/10μg, and Vss/F was 86.5 L for 5U/5μg and 94.0 L for 10U/10μg. The CL/F and Vss/F of lixisenatide were independent of dose (Table S3).

***Safety***

Three out of 20 patients treated with iGlarLixi 10U/10μg experienced mild nausea approximately 1.5 to 3 hours after administration. No patients experienced episodes of nausea under any of the other four study treatments. The patients in the iGlarLixi 10U/10μg group recovered within 5 hours after onset without any treatment. These episodes of nausea were considered by the investigator to be related to the study drug. No other TEAEs were reported during the study, and specifically no TEAEs or laboratory abnormalities related to pancreatitis, allergic or allergic-like reactions, or injection site reactions (Table S5).

Asymptomatic hypoglycemia with plasma glucose concentration ≤70 mg/dL was observed in 3 patients from 1.5 to 3 hours after administration of iGlarLixi 10U/10μg. No symptomatic hypoglycemia occurred during the study.
