# Supplementary material for: Effect of a single dose of insulin glargine/lixisenatide fixed ratio combination (iGlarLixi) on postprandial glucodynamic response in Japanese patients with type 2 diabetes mellitus: A phase I randomized trial
Source: Diabetes Obes Metab. 2019 May 24;21(8):2001–5. doi: 10.1111/dom.13757 (PMC6771557; doi:10.1111/dom.13757)
Supplement: Supplementary file 2 — TABLE S1. Patient characteristics. Table S2. Results for additional PD parameters of iGlarLixi. Table S3. Results for PK parameters of lixisenatide. Table S4. Dose proportionality assessment of lixisenatide (iGlarLixi 5 U/5 μg vs iGlarLixi 10 U/10 μg). Table S5. Summary of adverse events. Figure S1. Patient disposition. Figure S2. Changes in serum and plasma parameters for pharmacodynamics: insulin, C‐peptide and plasma glucagon. Figure S3. Changes in plasma concentration of lixisenatide for pharmacokinetics. [file DOM-21-2001-s002.pdf]

Supporting Information

Effect of single dose of insulin glargine/lixisenatide fixed ratio combination (iGlarLixi) on postprandial glucodynamic response in Japanese patients with type 2 diabetes mellitus: A phase 1 randomized trial

Contents

Supplementary Tables

TABLE S1 Patient characteristics — 2

TABLE S2 Results for additional PD parameters of iGlarLixi — 3

TABLE S3 Results for PK parameters of lixisenatide — 4

TABLE S4 Dose proportionality assessment of lixisenatide (iGlarLixi 5U/5µg vs. iGlarLixi 10U/10µg) — 5

TABLE S5 Summary of adverse events — 6

Supplementary Figure

FIGURE S1 Patient disposition — 7

FIGURE S2 Changes in serum and plasma parameters for pharmacodynamics: insulin, C-peptide and plasma glucagon — 8

FIGURE S3 Changes in plasma concentration of lixisenatide for pharmacokinetics — 9

Supplementary Tables

TABLE S1 Patient characteristics

	All patients (n=20)
Age, years	59.0 ± 8.5 (42 : 71)
Age group	
<65	15 (75.0)
≥65	5 (25.0)
Sex	
Male	17 (85.0)
Female	3 (15.0)
Weight at screening, kg	64.56 ± 12.51 (48.9 : 106.7)
BMI at screening, kg/m^2	23.77 ± 3.26 (19.3 : 33.5)
Creatinine at screening, µmol/L	62.90 ± 12.44 (40.7 : 89.3)
Duration of diabetes, years	6.91 ± 6.25
HbA1c value at screening, %	8.06 ± 0.69

Abbreviations: BMI, body mass index; HbA1c, hemoglobin A1c; SD, standard deviation.
Data are given as mean ± SD (Min : Max) or n (%) where indicated.

Table S2 Results for additional PD parameters of iGlarLixi

Parameter		iGlarLixi 5U/5µg (n=20)	iGlarLixi 10U/10µg (n=20)
PPG-AUC_{0-5}, mmol·hr/L	LS mean difference (SE) vs. placebo†	−14.88 (1.36)	−20.55 (1.36)
	95% CI	−17.62, −12.14	−23.29, −17.81
	P value	< 0.0001	< 0.0001
	LS mean difference (SE) vs. iGlarLixi 5U/5µg‡		−5.67 (1.36)
	95% CI		−8.40, −2.94
	P value		0.0001
	LS mean difference (SE) vs. insulin glargine§	−12.90 (1.36)	
	95% CI	−15.63, −10.17	
	P value	< 0.0001	
Serum insulin-AUC_{0-2}, pmol·hr/L	LS mean difference (SE) vs. placebo†	11.46 (16.63)	18.74 (16.68)
	95% CI	−21.89, 44.82	−14.72, 52.20
	P value	0.4936	0.2665
	LS mean difference (SE) vs. iGlarLixi 5U/5µg‡		7.27 (16.61)
	95% CI		−26.05, 40.60
	P value		0.6632
	LS mean difference (SE) vs. insulin glargine§	−38.75 (16.98)	
	95% CI	−72.77, −4.73	
	P value	0.0263	
Serum C-peptide-AUC_{0-2}, nmol·hr/L	LS mean difference (SE) vs. placebo†	−0.07 (0.09)	−0.33 (0.09)
	95% CI	−0.26, 0.11	−0.52, −0.15
	P value	0.4462	0.0007
	LS mean difference (SE) vs. iGlarLixi 5U/5µg‡		−0.26 (0.09)
	95% CI		−0.44, −0.08
	P value		0.0065
	LS mean difference (SE) vs. insulin glargine§	−0.13 (0.09)	
	95% CI	−0.32, 0.06	
	P value	0.1809	
Plasma glucagon-AUC_{0-2}, ng·hr/L	LS mean difference (SE) vs. placebo†	−23.88 (8.34)	−5.08 (8.48)
	95% CI	−40.63, −7.13	−22.10, 11.94
	P value	0.0061	0.5515
	LS mean difference (SE) vs. iGlarLixi 5U/5µg‡		18.80 (8.46)
	95% CI		1.82, 35.77
	P value		0.0306
	LS mean difference (SE) vs. insulin glargine§	−30.18 (8.34)	
	95% CI	−46.93, −13.42	
	P value	0.0007	

Abbreviations: iGlarLixi, insulin glargine and lixisenatide; PPG, postprandial plasma glucose; AUC, area under the curve; LS, least squares; SE, standard error; CI, confidence interval; PD, pharmacodynamics.
The linear mixed effects model that was used includes treatment group, sequence and treatment period as fixed effects, patient-within-sequence as a random effect, and the corresponding baseline all parameters value (−1 hr for time for PD) as a covariate.
†Difference: iGlarLixi 5U/5µg−placebo and iGlarLixi 10U/10µg−placebo, respectively
‡Difference: iGlarLixi 10U/10µg−iGlarLixi 5U/5µg
§Difference: iGlarLixi 5U/5µg−insulin glargine

Table S3 Results for PK parameters of lixisenatide

Parameter	iGlarLixi 5U/5µg (n=20)	iGlarLixi 10U/10µg (n=20)
C_{max}, pg/mL	51.6 ± 22.1<br>(46.4) [42.8]	110 ± 54.9<br>(97.2) [50.1]
t_{max}†, hr	2.50 (1.00 - 5.00)	2.00 (1.00 - 5.00)
$t_{1/2z}$, hr	2.50 ± 0.843<br>(2.38) [33.7]	2.73 ± 0.747<br>(2.65) [27.4]
AUC_{last}, pg·hr/mL	249 ± 99.8<br>(227) [40.1]	556 ± 202<br>(520) [36.3]
t_{last}†, hr	9.00 (5.00 - 10.00)	10.00 (10.00 - 10.00)
AUC_{0-2}, pg·hr/mL	61.2 ± 30.1<br>(52.3) [49.2]	129 ± 80.1<br>(109) [61.9]
AUC, pg·hr/mL	286 ± 101<br>(269) [35.2]‡	630 ± 225<br>(590) [35.7]‡
CL/F, L/hr	19.9 ± 7.99<br>(18.6) [40.0]‡	18.2 ± 7.76<br>(16.9) [42.6]‡
Vss/F, L	86.5 ± 36.0<br>(80.3) [41.6]‡	94.0 ± 55.6<br>(82.7) [59.2]‡

Abbreviations: iGlarLixi, insulin glargine and lixisenatide; C_{max}, maximum concentration; t_{max}, time to maximum concentration; AUC, area under the curve; SD, standard deviation; CV, coefficient of variation.
Data are given as mean ± SD (geometric mean) [CV%].
†Median (Min - Max)
‡n=17

TABLE S4 Dose proportionality assessment of lixisenatide (iGlarLixi 5U/5μg vs. iGlarLixi 10U/10μg)

Parameter	Estimate	95%CI
C_{max}	2.0934	1.6685, 2.6264
AUC_{last}	2.2884	1.8948, 2.7639
AUC	2.2178	1.7800, 2.7632

Abbreviations: AUC, area under the curve; CI, confidence interval.

Table S5 Summary of adverse events

	Placebo<br><br>(n = 20)	iGlarLixi<br>5U/5µg<br>(n=20)	iGlarLixi<br>10U/10µg<br>(n=20)	Insulin<br>glargine<br>(n=20)
Patients with any TEAE	0	0	3 (15.0)	0
Patients with any severe TEAE	0	0	0	0
Patients with any treatment emergent SAE	0	0	0	0
Patients with any TEAE leading to permanent treatment discontinuation	0	0	0	0

Abbreviations: iGlarLixi, insulin glargine and lixisenatide; TEAE, treatment emergent adverse event; SAE, serious adverse event.
Data are given as n (%) where indicated.
An adverse event is considered as treatment emergent if it occurred, worsened or became serious during treatment (injection with iGlarLixi 5U/5ug or 10U/10ug, placebo, or with insulin glargine, up to 24 hours after injection for each treatment period).

Supplementary Figure

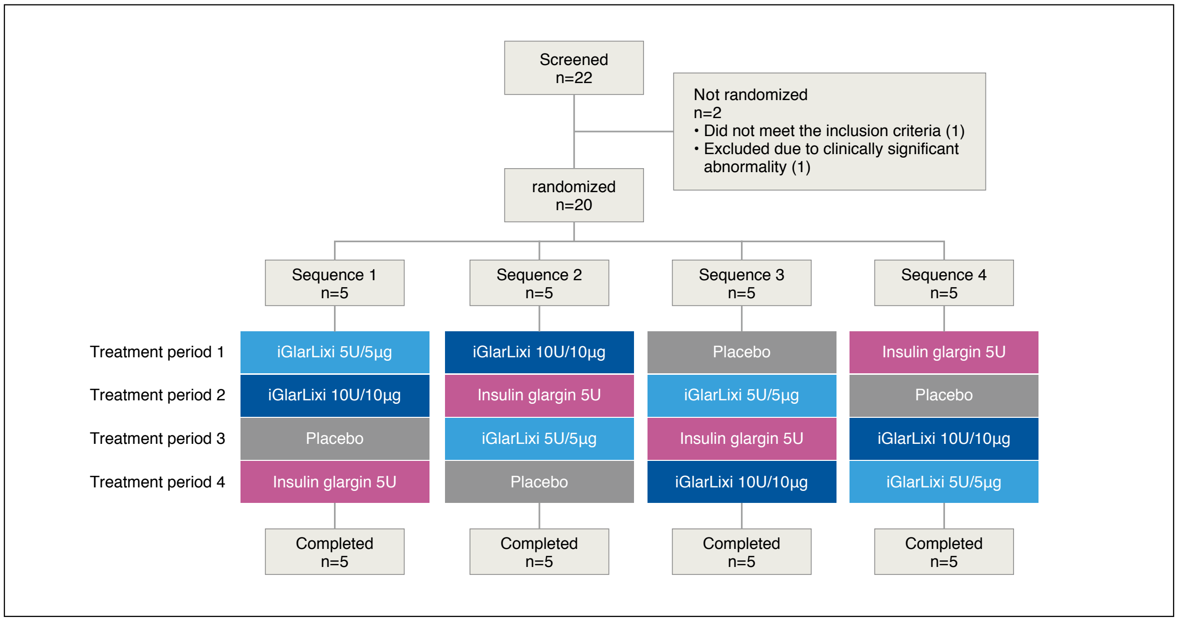

FIGURE S1 Patient disposition

Abbreviations: iGlarLixi, insulin glargine and lixisenatide.

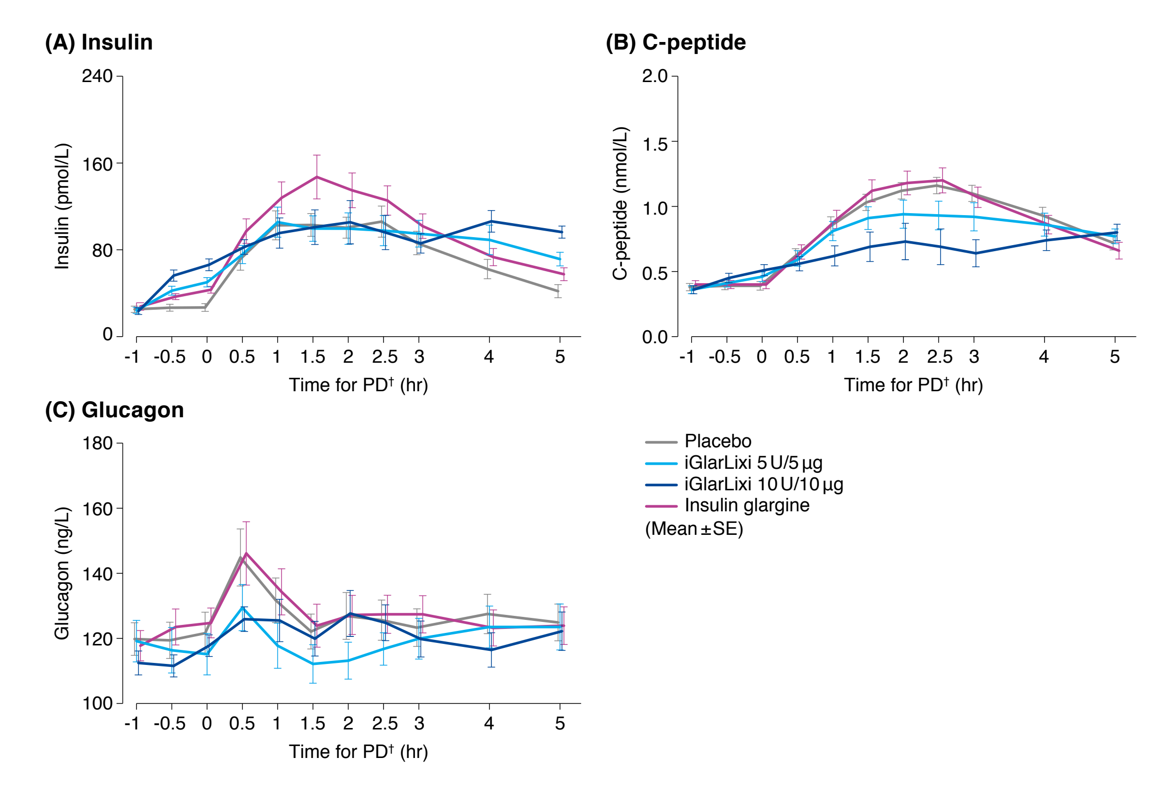

FIGURE S2 Changes in serum and plasma parameters for pharmacodynamics: insulin, C-peptide and plasma glucagon

Abbreviations: SE, standard error; PD, pharmacodynamics.

†Time for PD is shown in reference to the start of breakfast (0 hr in the figure). Breakfast was served 1 hr after administration of the study drug.

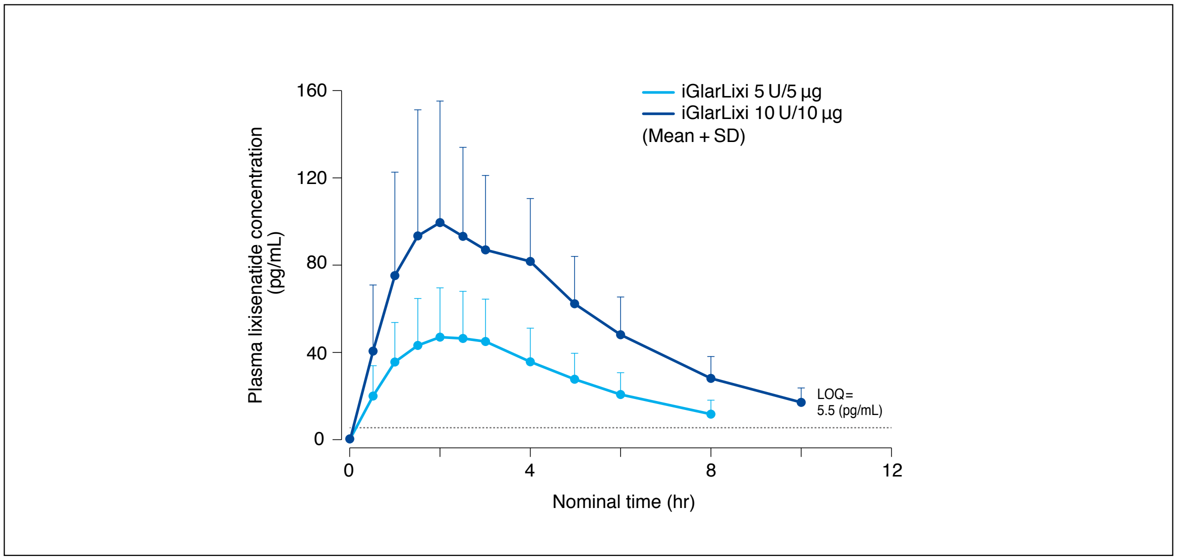

FIGURE S3 Changes in plasma concentration of lixisenatide for pharmacokinetics

Abbreviations: SD, standard deviation; LOQ, limit of quantification.
